# Dataset of phase I and II immunotherapy clinical trials used for a meta-analysis to assess the role of biomarkers in treatment outcomes in diverse cancers

**DOI:** 10.1016/j.dib.2023.109698

**Published:** 2023-10-16

**Authors:** Elena Fountzilas, Henry Hiep Vo, Peter Mueller, Razelle Kurzrock, Apostolia-Maria Tsimberidou

**Affiliations:** a Department of Medical Oncology, St Luke's Clinic, Panorama 552 36, Thessaloniki, Greece; b European University Cyprus, 6 Diogenous Str., Egkomi, 2404, Nicosia, Cyprus; c The University of Texas MD Anderson Cancer Center, Department of Investigational Cancer Therapeutics, 1515 Holcombe Blvd, Houston, TX 77030, USA; d Department of Statistics and Data Science, The University of Texas at Austin, 105 E 24th St D9800, Austin, TX 78705, USA; e WIN Consortium for Precision Medicine, Albert Thuret 24, 94550 Chevilly-Larue, France; f Medical College of Wisconsin, 8701 W Watertown Plank Rd, Milwaukee, WI 53226, USA

**Keywords:** Biomarker, Clinical trial, Immunotherapy, Meta-analysis, MSI, Multivariate analysis, PD-L1

## Abstract

We performed a literature search in PubMed to identify phase I/II clinical trials with immunotherapy drugs approved by the Food and Drug Administration (labeled, off-label, and/or combined with investigational immune checkpoint inhibitors or other treatment modalities) from 2018 to 2020. We used the following key words: clinical trials, phase 1, Phase 2; and the following filters: cancer, humans; and selected the checkpoint inhibitors that had been approved by the FDA by March 2021, i.e., “pembrolizumab”, “nivolumab”, “atezolizumab”, “durvalumab”, “cemiplimab”, “avelumab”, and “ipilimumab. Clinical trials with their checkpoint inhibitors as in their labeled indications, off-label use or their combinations with investigational immune checkpoint inhibitors or other treatment modalities were included. Studies describing supportive care or locoregional treatments; cellular, viral, or vaccine therapy; studies in the adjuvant or neoadjuvant setting; and pediatric studies were excluded. Overall, 173 articles reporting on relevant studies were identified. Using these articles, we compiled a data file of study-specific covariates for each study. We recorded the immunotherapeutic agent, tumor type and biomarker, and clinical outcomes (objective response rate and median values [point estimate] and confidence intervals for progression-free survival and overall survival. Using these data, we carried out meta-analyses for the three outcomes and meta-regression on study-specific covariates. The same data could be used for any alternative implementation of meta-analysis and meta-regression, using more structured inference models reflecting different levels of dependence based on the available study-specific covariates.

Specifications Table

**Table:** 

Subject	Health and medical sciences
Specific subject area	Predictive biomarkers for immune checkpoint inhibitors (ICIs)
Type of data	Phase I and II clinical trial data
How the data were acquired	A PubMed search for phase I/II clinical trials with Food and Drug Administration (FDA)-approved ICIs (2018 to 2020) identified 173 articles reporting on relevant studies. Data collection was carried out by manual review of articles. Study-specific covariates and outcome data (ORR, PFS, and OS) were recorded. Confidence intervals were recorded for PFS and OS, if available. Selected articles reported summaries stratified by biomarker status for multiple biomarkers, while other reported summaries stratified by patient subgroups. Data were recorded for each biomarker or subgroup separately.
Data format	Raw
Description of data collection	We compiled a data file that included the following information: number of patients; age (median, range); tumor type; immunotherapy agent; monotherapy or combination therapy; biomarker and use (prospective or not); number of patients in the marker-positive and marker-negative cohorts; line of therapy; prior therapies; biomarker association with resistance or sensitivity to immunotherapy. Details in Figure 1 (PRISMA diagram).
Data source location	The raw data source are the articles on phase I/II clinical trials using ICIs identified in the PubMed search. The data were not acquired privately from source authors. Therefore, no permission to include these data as a supplement should be obtained.
Data accessibility	Repository name: Figshare Direct URL to data: https://figshare.com/articles/dataset/MetaAnalysisDataIO_3_xlsx/23613489 All primary data sources are listed in this spreadsheet.
Related research article	E. Fountzilas, H. H. Vo, P. Mueller, R. Kurzrock, A. M. Tsimberidou, Correlation between biomarkers and treatment outcomes in diverse cancers: a meta-analysis of phase I and II immunotherapy clinical trials. Eur J Cancer. 2023 May 22;189:112927. doi:10.1016/j.ejca.2023.05.015 https://pubmed.ncbi.nlm.nih.gov/37364526/

## 1 Value of the Data

• Many immuno-oncology trials are conducted without biomarker selection. We performed a meta-analysis of phase I/II clinical trials evaluating immune checkpoint inhibitors (ICIs) to determine the association between biomarkers and clinical outcomes, if any.
• This meta-analysis of data from phase I/II clinical trials with immunotherapy drugs published from 2018 to 2020 showed that the immune-related biomarker-positive cohort had higher response rates and longer progression-free and overall survival after immune checkpoint blockade compared with the biomarker-negative cohort.
• This is a unique database that provides all the raw data that were meticulously collected and recorded. To our knowledge, this is the first and the only publicly available database summarizing the results of phase I and II clinical trials with checkpoint inhibitors. New data and new publications can emerge from the analysis of this valuable resource.
• These data are a source of published clinical outcomes with and without biomarker use for patient selection. Raw data have not been previously published and can be valuable to the research community.
• The data file includes study-specific covariates for each study, that describes the immunotherapeutic agent, tumor type and biomarker, and clinical outcomes (objective response rate and median values [point estimate] and confidence intervals for progression-free survival and overall survival).
• The same data could be used for any alternative implementation of meta-analysis and meta-regression, using more structured inference models reflecting different levels of dependence based on the available study-specific covariates. Other investigators, healthcare professionals, biotechnical industry, regulatory agencies as well as patients could exploit the database.
• These results demonstrate the significance of the use of immune-related biomarkers for the selection of patients with diverse tumor types who will participate in clinical trials evaluating ICIs. Prospective clinical trials need to implement the use of composite biomarkers by incorporating genomic, transcriptomic, and immune profiles, host pharmaco-genome, and other factors.

## 2 Data Description

The spreadsheet shared at the indicated anonymous ftp site provides the complete secondary data used in the meta-analysis. In the spreadsheet, each line corresponds to one cohort of patients, with some items reported separately for marker-positive and marker-negative patients in the indicated cohort. In many cases, a single study includes data on multiple cohorts defined by different disease subtypes or by different biomarkers. In such cases, multiple lines in the spreadsheet are used to summarize the study. The headers in line 1 of the spreadsheet indicate the reported information.

Additionally, the analysis used a derived variables hazard ratio (HR) and weights (w). For the HR we used a point estimate as the ratio of median event times (this would be exact under exponential sampling models). For the study-specific weights we used sum of the inverse sample sizes (of marker-positive and marker-negative cohorts).

Details of the data entries in the spreadsheet are reported in Table 1.

**Table 1:** Columns in the data spreadsheet.

Column	Column header	Reported information	Comments
A	New numbering MASTER	Running index of cohort	(For internal use only)
B	Numbering new with …	Running index of cohort	(For internal use only)
C	Trial numbering	Running index of study	
D	PMID	PubMed ID of original article	
E	Phase	Phase of study	
F	Treatment	= being studied	
G	Monotherapy	Indicator for mono- or combination immunotherapy	
H	Combination with	In the case of combination therapy	
I	Specify combination	Specify agent/treatment modality that was combined with immunotherapy	
J	Title	Title of original article	
K	Authors	Authors of original article	
L	Journal	Journal publishing original article	
M	Publication data	Publication date of original article	
N	Abstract	Abstract of original article	
O	Link	URL for original article	
P	Tumor type	Tumor type(s) included in original article	
Q	No of patients	Total number of patients included in the study	
R	Gender	If there is a restriction by gender	
S	Age, median	Median age of all patients enrolled in the study	
T	Age, range	Range of ages of all patients	
U	Timing of biomarker analysis - only approved immune related biomarkers	Retrospective or prospective nature of the analysis of approved immune-related markers	
V	Timing of biomarker analysis – other biomarkers	Retrospective or prospective nature of the analysis of other immune-related markers	
X	Patient selection [“based on biomarkers”]?	Indicator of whether the study design included patient selection based on biomarkers	
W	Timing of biomarker analysis	Retrospective or prospective nature of the analysis or both	
Y	Specify biomarker	If “yes” on X, specify biomarker	
Z	Correlative studies	Indicator of whether the study design included correlative studies of biomarkers	
AA	Specify biomarker	If “yes” on Z, specify biomarker	Using separate lines for multiple markers
AB	No. of pts with biomarker	No. of pts for whom biomarker could be evaluated	
AC	Biomarker data	Miscellaneous comments	
AD	Comments	Miscellaneous comments	
AE	Prior treatment allowed	Whether eligibility for the study included patients with prior treatments	
AF	Line of immunotherapy	In case of “yes” for AE	
AG	Sequence of IO in chemotherapy combination trials	In case of “yes” for AE	
AH	Number of prior therapies allowed	In case of “yes” for AE	
AI	Tumor markers associated with resistance to IO	Whether the selected marker is associated with resistance to immunotherapy	
AJ	Tumor markers associated with sensitivity to IO	Whether the selected marker is associated with sensitivity to immunotherapy	
AK	Was prior immunotherapy allowed		
AL	No. of prospective biomarkers based on which patient selection was performed		
AM	No. of retrospective biomarkers in correlative analysis		
AN	Comments		
AO	Technology used	Methodology of biomarker assessment	
AP	Cut-off	If relevant for AO	
	**Data summaries for the entire patient cohort:**		
AQ	All patients ORR %	Objective response rate for entire patient cohort, as percent	
AR	All patients ORR N	Same as AR, as fraction	
AS	All patients PFS	Median progression free survival: point estimate (C.I)	C.I is 95% C.I unless otherwise indicated
AT	All patients OS	Same for overall survival	
	**Data summaries by biomarker status**		
AU	Number of patients with positive biomarker	Number of marker-positive patients	
AV	Number of patients with negative biomarker	Number of marker-negative patients	
AW	Biomarker positive patients ORR%	Objective response rate for marker-positive patients, in percent	
AX	Biomarker positive ORR	Same as fraction	
AY	Biomarker negative patients ORR%	Objective response rate for marker-negative patients, in percent	
AZ	Biomarker negative ORR%	Same as fraction	
BA	Statistical significance between ORR based on investigators	Whether article reports statistically significant difference of ORR between marker-positive and -negative cohorts	
BB	Biomarker positive PFS (Md, Lo, Hi)	Same as AT for marker-positive patients	
BC	Biomarker negative PFS (Md, Lo, Hi)	Same as AT for marker-negative patients	
BD	Biomarker positive OS (Md, Lo, Hi)	Same as AU for marker-positive patients	
BE	Biomarker negative OS (Md, Lo, Hi)	Same as AU for marker-negative patients	

## 3 Experimental Design, Materials and Methods

**Fig. 1:**
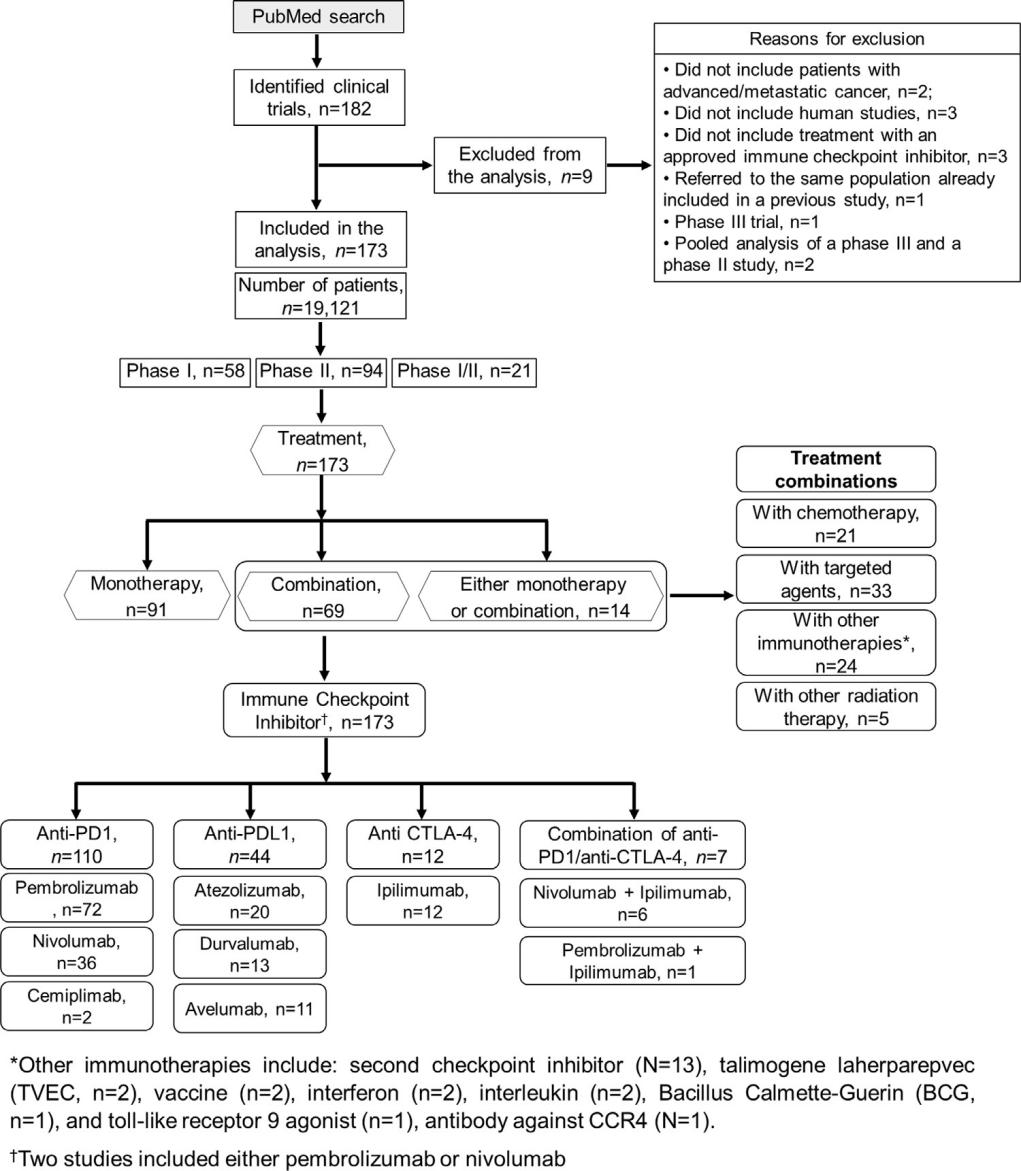
PRISMA diagram. Figure reproduced with permission from Fountzilas et al, Eur J Cancer. 2023 May 22;189:112927, DOI: 10.1016/j.ejca.2023.05.015. Copyright 2023, Eur J Cancer.

Each of the articles identified in the PubMed search was reviewed by one of the authors. The search included the following terms: clinical trials, phase 1, Phase 2,” and publication dates from January 1, 2018, to December 31, 2020. The following filters were also used: “cancer”; and “humans”. The checkpoint inhibitors that had been approved by the FDA by March 2021, i.e., “pembrolizumab”, “nivolumab”, “atezolizumab”, “durvalumab”, “cemiplimab”, “avelumab”, and “ipilimumab” were selected in the search. Clinical trials with their checkpoint inhibitors as in their labeled indications, off-label use or their combinations with investigational ICIs or other treatment modalities were included. Studies describing supportive care or locoregional treatments; cellular, viral, or vaccine therapy; studies in the adjuvant or neoadjuvant setting; and pediatric studies were excluded (Fig. 1).

We first verified that the article did indeed report on a phase I or II immuno-oncology trial and that it included outcomes stratified into biomarker-positive and -negative cohorts. We then recorded all study-specific covariates and the outcome summaries. Before proceeding with the desired meta-analysis we carried out a test for homogeneity (fixed effects only), to use a random effects model only if the null hypothesis of common fixed effects could be rejected [1]. We used the method of DerSimonian-Laird [2] to estimate residual heterogeneity. For all three endpoints, ORR, PFS and OS, homogeneity was rejected with *p* < 0.0001.

For ORR, we recorded the number of patients and number of overall responses by marker status. Analyses of ORR were based on log ORs. For the event time outcomes, we recorded point estimates and 95% confidence intervals for the median PFS and OS durations, where available. When the upper limit of the confidence interval was not available, we recorded it as N/R (not reached). In some cases, we referred to Kaplan-Meier plots to identify sample sizes and estimates. Results for event times (PFS and OS) were based on differences of log median event times for biomarker-negative versus biomarker-positive groups [3]. For each study we evaluated study-specific variance as the sum of inverse sample sizes, as this was readily available for all studies in the meta-analysis [4]. Tests and coding of variables were performed as previously published. Statistical significance was defined as *p* < 0.05, and highly significant was defined as *p* < 0.0001.

All reported inference was performed in the R statistical software environment [5]. We used implementation of meta-analysis in the R packages metaphor [6], meta [7] and mvmeta [8]. Summaries are displayed as forest plots [9]. All event times were converted to months, if needed.

Potential biases were addressed by (i) inspection of a funnel and trim-and-fill plot; (ii) by carrying out meta-regression on agent, tumor type, monotherapy vs. combination therapy, line of therapy, and tumor types [10], [11], [12]. The trim-and-fill method was proposed in Duval and Tweedie [11,12]. It estimates the number of studies missing from a meta-analysis due to lack of significant results. We implement the method using the R package metafor [6]. Meta-regression describes how different study characteristics impact the overall treatment effect being reported in the meta-analysis. We use an implementation of meta-regression from the R package meta [13]. Neither of the two methods identified evidence for systematic biases. Finally, we note that the results in the related research article are based on the data file with one study erroneously included in duplicate (PMID: 30515672). The duplication is removed in the shared data set. Repetition of the analysis, after excluding the duplicate study, did not alter study results.

## Ethics Statements

The work was carried out on data from published studies and did not involve animals or human subjects.

## CRediT authorship contribution statement

**Elena Fountzilas:** Data curation, Investigation, Methodology, Validation, Writing – original draft, Writing – review & editing. **Henry Hiep Vo:** Data curation, Investigation, Methodology, Validation, Writing – original draft, Writing – review & editing. **Peter Mueller:** Formal analysis, Funding acquisition, Investigation, Methodology, Software, Validation, Writing – original draft, Writing – review & editing. **Razelle Kurzrock:** Conceptualization, Funding acquisition, Investigation, Methodology, Validation, Writing – original draft, Writing – review & editing. **Apostolia-Maria Tsimberidou:** Conceptualization, Funding acquisition, Investigation, Methodology, Validation, Writing – original draft, Writing – review & editing.

## Data Availability

MetaAnalysisDataIO (Original data) (FIGSHARE).
